# Impact of Positive Feedback on Antimicrobial Stewardship in a Pediatric Intensive Care Unit: A Quality Improvement Project

**DOI:** 10.1097/pq9.0000000000000206

**Published:** 2019-08-30

**Authors:** Alison S. Jones, Rhian E. Isaac, Katie L. Price, Adrian C. Plunkett

**Affiliations:** From the Birmingham Women’s and Children’s NHS Foundation Trust, Birmingham, United Kingdom.

## Abstract

We hypothesized that antimicrobial stewardship (AMS) could be enhanced through positive feedback for the behaviors of healthcare professionals. This project aimed to reduce antimicrobial consumption in a Pediatric Intensive Care Unit (PICU) by >5%, with secondary aims to reduce broad-spectrum antimicrobial consumption, and processes related to AMS. Learning from Excellence is a positive feedback initiative conceptualized at our institution.

**Methods:**

This project took place over 12 months (April 2017–March 2018) in a 31-bedded PICU. We identified and measured processes about AMS daily. Healthcare professionals, achieving success in these processes, received positive feedback via Learning from Excellence, during a 6 months intervention period. Selected reports were followed with appreciative inquiry interviews to reinforce positive feedback. We calculated antimicrobial consumption data from existing databases (antimicrobial doses dispensed divided by PICU bed-days). Health Care-Associated Infection (HCAI) rates were included as a balancing measure.

**Results:**

Antimicrobial consumption was 6.5% lower during the intervention period compared with the matching period from the previous year. We reduced broad-spectrum antimicrobial (meropenem) consumption by 17.6%. Improvements in processes were mixed: a daily review of antimicrobials and documentation of antimicrobial prescription and administration significantly improved. Other processes failed to improve. HCAI rates did not change.

**Conclusions:**

Positive feedback can be used as a QI intervention to improve processes around AMS. This intervention may contribute to a reduction in antimicrobial consumption. Not all processes are impacted equally, and there may be a “dose-response” effect. Further evaluation would benefit from a trial study design in other settings.

## Abstract 2

Supplemental Digital Content is available in the text.

## INTRODUCTION

### Problem Description

The increasing prevalence of antimicrobial resistance (AMR) is recognized globally as a major threat to healthcare.^1^ A key driver in AMR is exposure to antimicrobial therapy,^2^ a significant proportion of which is inappropriate.^3^ Safe reductions in antimicrobial exposure are difficult to achieve due to a lack of rapid, reliable diagnostic tests for infection and sepsis,^1^ and a competing drive to rapidly and aggressively treat sepsis.^4^ The rivalry between the short-term gain of timely treatment of acute sepsis, and the longer-term gain of reducing antimicrobial exposure is played out daily in the Pediatric Intensive Care Unit (PICU), where the prevalence of infection and sepsis is high,^5^ and an estimated 40%–80% of patients in the PICU receive antimicrobials.^6^ Within the UK National Health Service (NHS), there is a national requirement to reduce antimicrobial consumption year on year. NHS organizations are responsible for implementing local quality improvement interventions to reduce antimicrobial consumption. This project is a departmental response to this national and global issue.

### Available Knowledge and Rationale

Antimicrobial stewardship (AMS) addresses all the elements of antimicrobial therapy,^7^ including initiation of appropriate treatment; timely administration; regular review; and safe cessation of antimicrobials. There is no recognized, optimal strategy for AMS, although multiple processes underpinning AMS are acknowledged in the literature.^8^ Many AMS processes are embedded in the behaviors of healthcare professionals (HCP). For example, prescribing practice is central to AMS initiatives, including the “start smart and focus” initiative, from the UK Department of Health;^7^ “optimizing prescribing through stewardship” is also identified as a key area for action in the UK 5-year AMS strategy.^9^

The prevailing approach to influencing prescribing practice, and other HCP behaviors, is to highlight errors and deficits; that is, to influence behavior through negative feedback. While this approach may be successful, it overlooks the opportunity to learn from positive feedback: a potentially highly effective stimulus for learning and improved motivation.^10^ Learning from Excellence (LfE) is a novel initiative in healthcare which aims to realize the benefit of positive feedback in a healthcare setting.^11^

The LfE system formed the basis of the QI interventions in this project. The working title of the project was PRAISe: Positive Reporting and Appreciative Inquiry (AI) in Sepsis and Stewardship.

### Specific Aims

We hypothesized that positive feedback for behaviors related to AMS processes would impact antimicrobial consumption. The primary aim was to reduce antimicrobial consumption (antimicrobial doses per PICU bed-day, including all patients in PICU) by >5% during the 6-month intervention (July 2017–December 2017), compared with the equivalent time-period for the previous year. Secondary aims were to reduce broad-spectrum antimicrobial (meropenem) consumption in the same time-frame and to improve processes related to AMS during the project.

## METHODS

### Context

#### The Environment.

Birmingham Children’s Hospital PICU comprises 31 bed-spaces and admits ~1,400 cases per year from multiple medical and surgical specialties. The multidisciplinary workforce includes 370 staff members.

#### Antimicrobial Management—Institutional.

Within our institution, a multi-disciplinary antimicrobial management committee oversees AMS across the organization and responds to the mandatory national audit. Each department in the organization is represented on this committee. AMS interventions at the departmental level are the prerogative of individual departments. Our project did not influence the actions of the committee–this project was over and above the regular activities of the antimicrobial management committee.

#### Antimicrobial Management—in PICU.

Clinical decision-making occurs continuously in the clinical area, and during thrice daily ward rounds, led by the PICU consultant. Continuity of PICU consultant is maintained 5 days per week (Monday to Friday day-time), with on-call cover from other PICU consultants outside these hours.

Additional antimicrobial decision-making occurs during daily meetings between the PICU consultant, microbiologist, and antimicrobial pharmacist. Institutional guidelines guide antimicrobial decision-making. Prescriptions are hand-written on paper charts in designated prescribing areas. Doctors in training and advanced nurse practitioners document the majority of prescriptions.

#### Antimicrobial Restrictions.

During the timeline of the project, piperacillin-tazobactam (“tazocin”) was restricted across the organization. Restriction of piperacillin-tazobactam is associated with a theoretical risk of increased consumption of similar broad-spectrum antimicrobials, eg, meropenem. For this reason, we chose meropenem consumption as the most appropriate broad-spectrum antimicrobial in our formulary to measure, thus avoiding confounding effects of drug shortage on the project outcome.

#### Learning from Excellence.

LfE was conceptualized and implemented in our unit in 2014.^11^ LfE is a staff peer-reporting system with 2 aims: to improve quality of care by examining what is working well in the system; and to provide positive feedback to staff. The system consists of an online reporting form, available to all staff. Excellence reports (referred to hereafter as LfE reports) are completed voluntarily in response to episodes of good practice; the reports are delivered electronically to cited colleagues (typically within 24 hours), thereby providing positive feedback.

The LfE team reviews all reports. Selected reports judged to contain significant learning potential, are investigated in more detail through semistructured conversations using AI methodology (see Supplemental Digital Content at http://links.lww.com/PQ9/A132 for Table S1). AI is an action research framework, used to identify and enhance the conditions that allow excellence to flourish.^12^ A baseline reporting rate of 50–100 LfE reports per month was present before the commencement of the project. PICU staff were, therefore, familiar with LfE at the start of the project.

#### Incident Reporting System.

Errors and other adverse incidents were reported through an established organization-wide incident reporting system. The 2 reporting systems were separate and accessed by separate electronic forms, available on the hospital intranet.

#### Project Team and Interventions.

**Fig. 1.**
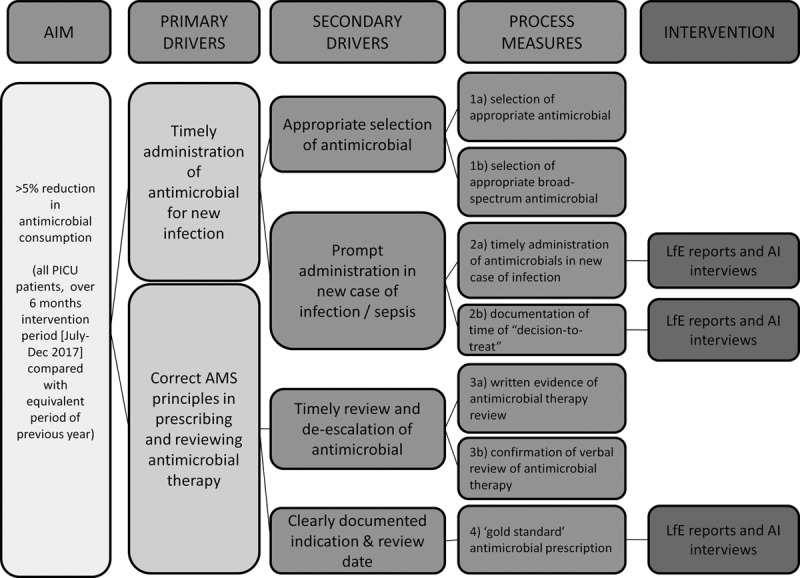
Driver diagram.

We convened a project team at the project set-up. It consisted of medical, nursing, pharmacy, and project management personnel. Team meetings were scheduled weekly throughout the project. A driver diagram was used to identify the key processes of AMS and areas for intervention (Fig. 1).

The project period was divided into 3 phases:

- Pre-intervention phase (3 months: April–June 2017): the collection of baseline data.
- Intervention phase (6 months: July–December 2017): measurement of the intervention effect.
- Post-intervention phase (3 months: January–March 2018): measurement of residual effects of the intervention.

We adopted a strategy of continuous improvement during the intervention period. Interventions were adapted from the LfE system, and comprised 2 forms of positive feedback:

1. Individual HCPs who achieved success in 1 of the 2 intervention areas (ie, gold-standard prescribing practice, or administration of timely new antibiotics), received positive feedback via a LfE report with a description of what they had achieved, and why this was helpful for AMS. Reports were filed using the LfE system. Thus, recipients received the notification via email, typically within 24 hours of the action. The reports were entered on the LfE system by members of the project team. An example report is shown in Supplemental Digital Content at http://links.lww.com/PQ9/A131 for Figure S1.
2. Selected LfE reports were followed with an appreciative interview, to enhance positive feedback, and to gather improvement ideas. The interview structure was an AI protocol, adapted to allow the interview to take place within 10–15 minutes. We conducted a purposive sampling of interview subjects to ensure even distribution of participation throughout the workforce. There were no exclusion criteria for selection, but repeat interviews with HCPs were avoided. The interview schedule is shown in Supplemental Digital Content at http://links.lww.com/PQ9/A132 for Table S1.

In addition to the primary effect of positive reinforcement, the AI interviews were also utilized to generate further improvement ideas. We reviewed these interviews in project meetings, and selected interventions were implemented throughout the project. These interventions are listed in Supplemental Digital Content at http://links.lww.com/PQ9/A132 for Table S2, and respective dates of implementation are annotated on the relevant statistical process control (SPC) charts in the results section.

#### Measurement.

Definitions of measures, frequency, and method of collection are detailed in Table 1. We collected data from the clinical area (process measures) and electronic databases (outcome measures). A sample of (4–8) PICU beds was reviewed each day, ensuring that data were obtained from all 31 beds at least once per week.

**Table 1.**
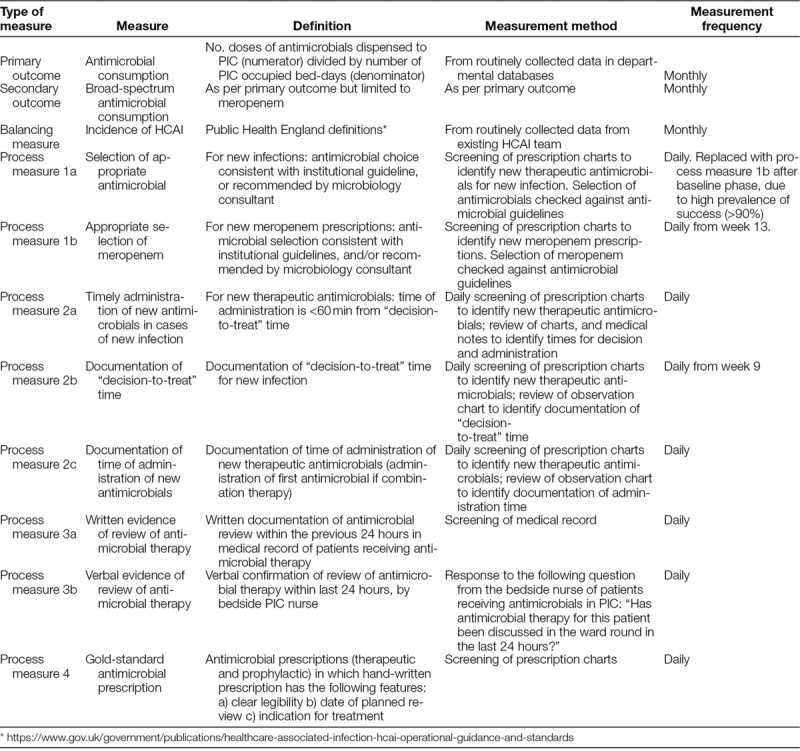
Measures

#### Study of the Measures and Statistical Methods.

Two statistical methods and time-frames were used in this project:

*Outcome Measure*. We compared the consumption data during the intervention period (July–December 2017) with the matching period from the previous year (July–December 2016), in recognition of the significant seasonal variation of case-mix in PICU.^13^ We calculated consumption data from the 2 cohorts as the number of antimicrobial doses dispensed per PICU bed-day (using aggregated data from pharmacy and PICU activity records). Analysis was restricted to commonly-used antimicrobials. (We excluded antiviral and antifungal therapy due to the irregularity of use.) The full list of antimicrobials is detailed in Supplemental Digital Content at http://links.lww.com/PQ9/A132 for Table S3.

*Process Measures*. Process measures were recorded throughout the 3 phases of the project (ie, April 2017–March 2018). We recorded data as binary outcomes (yes/no), and monitored weekly with SPC charts. Centerlines were reset if 8 consecutive values fell above or below the centerline. The balancing measure (HCAI rate) was collected as monthly aggregate data and presented in SPC chart format.

#### Ethical Approval

The project was designated as service improvement by the institutional Research, Development, and Innovation Department. Therefore, formal ethical approval was not required.

## RESULTS

### Project Activity

We screened a total of 1,968 bed-spaces during the project (April 2017–March 2018): a mean of 39 bed-spaces per week (range 25–54). During the intervention phase (July-December 2017), we generated 554 excellence reports and conducted 76 AI interviews.

### Antimicrobial Consumption

There was a reduction in total antimicrobial consumption, measured as antimicrobial doses per PICU bed-day: 2.15 versus 2.01 doses per bed-day, a relative reduction of 6.5%. The consumption of meropenem decreased from 0.37 to 0.30 doses per PICU bed-day, a relative reduction of 17.6%. Cohort characteristics (in terms of age, sex, length of stay, elective admission rate, and mortality) between the 2 periods are detailed in Table 2.

**Table 2.**
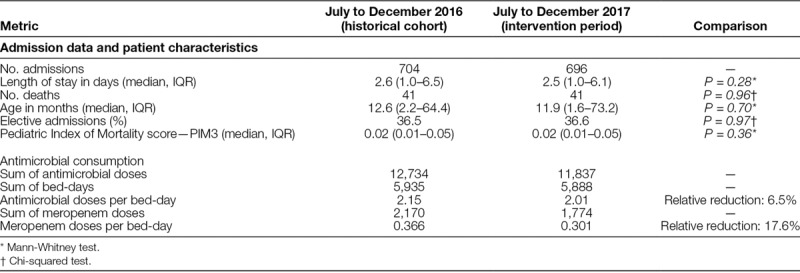
Comparison of Cohorts and Antimicrobial Consumption

#### Balancing Measure.

HCAI rates did not significantly change during the study period (see Supplemental Digital Content at http://links.lww.com/PQ9/A131 for Figure S2).

### Process Measures

#### Process Measure 1.

1a: During the pre-intervention phase, the choice of antimicrobial was appropriate in >95% of prescriptions. 1b: Meropenem as a new antibiotic was appropriately selected on 87% of occasions throughout the project with no special cause variation (see Supplemental Digital Content at http://links.lww.com/PQ9/A131 for Figure S3)

#### Process Measure 2.

**Fig. 2.**
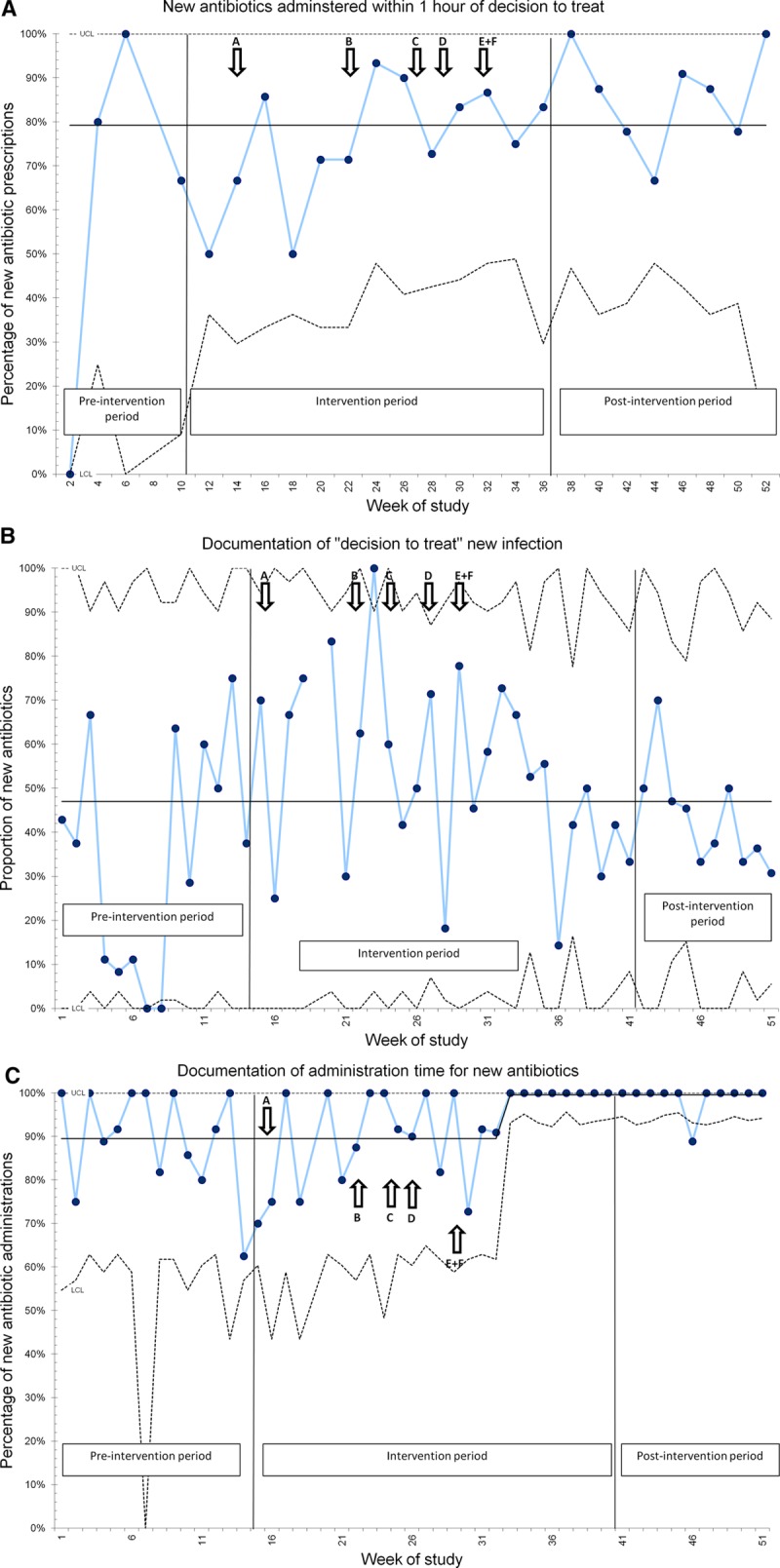
SPC charts for process measures 2a, 2b, and 2c. Additional interventions generated from AI interviews are labeled A–F: see Supplemental Digital Content at http://links.lww.com/PQ9/A132 for Table S2.

2a: Complete data for “decision-to-treat” time and administration-time for new antimicrobials was available for 217 antimicrobial courses (36% of all new antimicrobial courses captured during the study). Within this cohort, 79% of new antimicrobials were administered ≤1 hour of “decision-to-treat” time, with no special cause variation detected. 2b and 2c: Rate of documentation of “decision-to-treat” time was 47% throughout the project, with no improvement despite intervention; however, there was a significant improvement in documentation of administration time toward the end of the project; from 90% to 100%. SPC charts for processes 2a, 2b, and 2c are shown in Figure 2.

#### Process Measure 3.

**Fig. 3.**
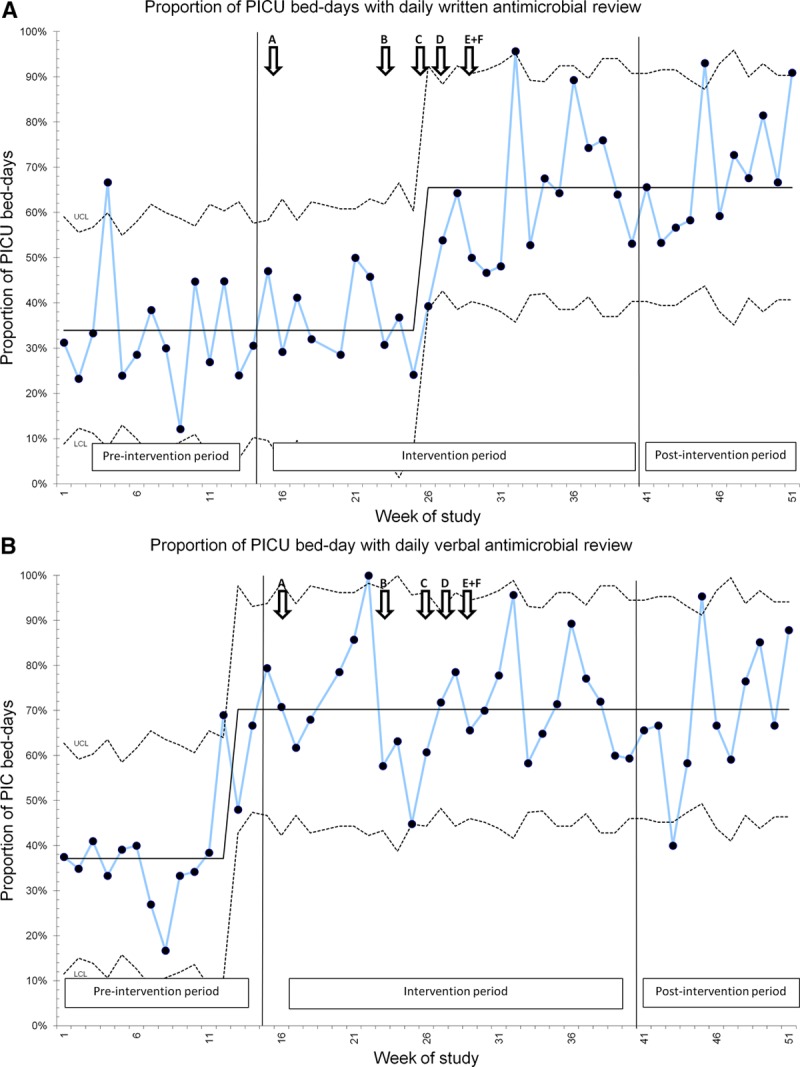
SPC charts for process measures 3a and 3b. Additional interventions generated from AI interviews are labeled A–F: see Supplemental Digital Content at http://links.lww.com/PQ9/A132 for Table S2.

Written and verbal confirmation of daily review of antimicrobials improved significantly during the study period from 36% to 66% and from 38% to 65%, respectively (Fig. 3).

#### Process Measure 4.

**Fig. 4.**
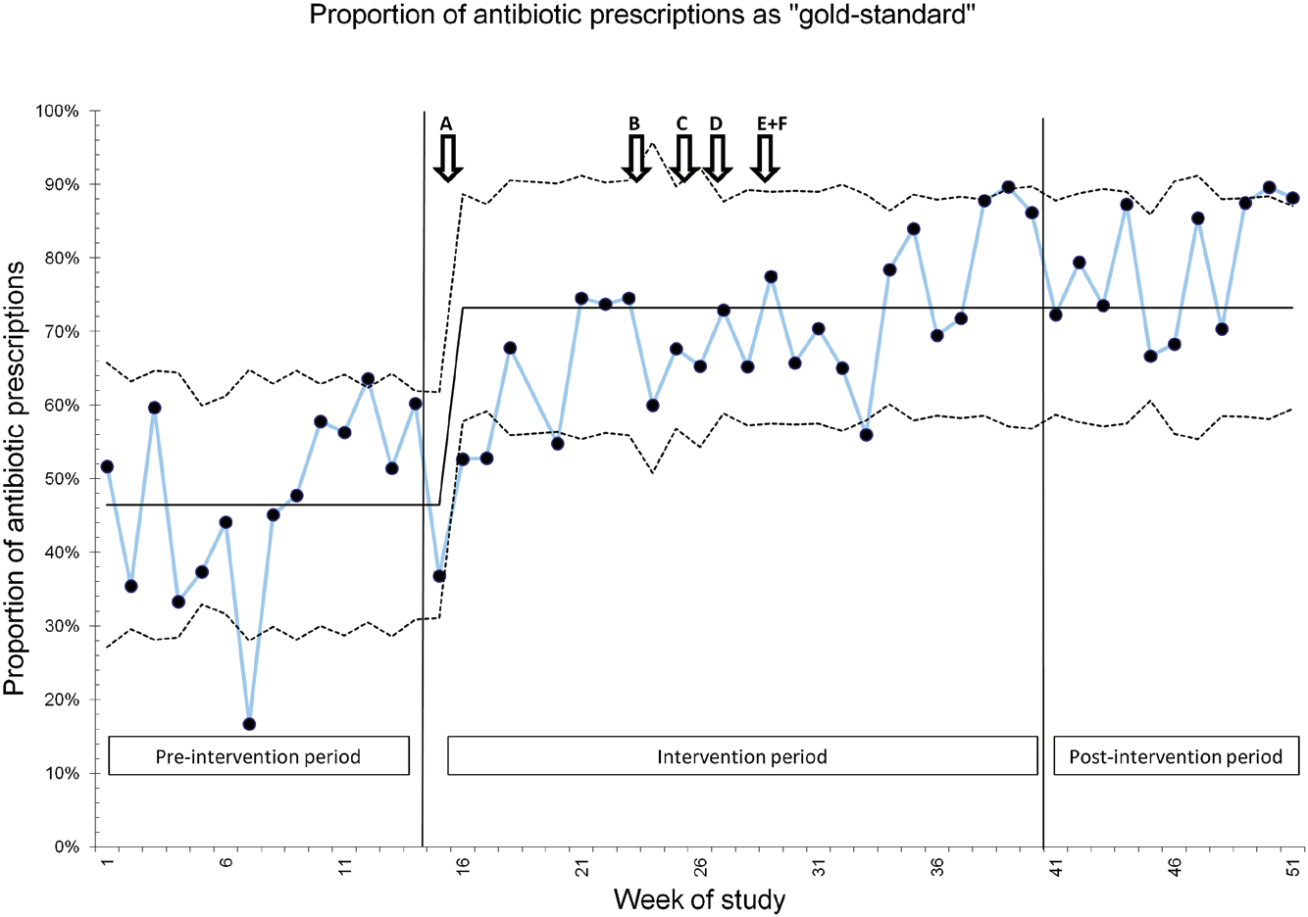
SPC chart for process measure 4. Additional interventions generated from AI interviews are labeled A–F: see Supplemental Digital Content at http://links.lww.com/PQ9/A132 for Table S2.

“Gold standard” antimicrobial prescribing improved significantly during the study period, from 46% to 73% (Fig. 4).

Raw data from all process measures, including numerator and denominator data, are included in Supplemental Digital Content at http://links.lww.com/PQ9/A132 for Table S4–S11.

## DISCUSSION

Consistent with the aims of the project, antimicrobial consumption was >5% lower during the intervention period compared with the matching period the previous year, and there was a greater reduction in broad-spectrum antimicrobial consumption. There was no measurable change in the rate of HCAI, indicating that the reduction in antimicrobial consumption was not associated with a rise in new infections.

We based this project was based on the supposition that positive feedback for behaviors related to AMS would lead to improvements in those behaviors, and this, in turn, would lead to reductions in antimicrobial consumption. This causal chain relies on several assumptions, and the improvements seen in this project may not be wholly attributable to the interventions. However, the findings suggest that reinforcing good practice through positive feedback may be a valid QI intervention.

Several processes improved throughout the project, but the effects were not universal. There were mixed effects in areas of direct intervention: “gold-standard” prescribing improved, whereas documentation of “decision-to-treat” time failed to improve. This dichotomy may reflect characteristics of the individual processes. For example, prescribing occurs multiple times per day and is carried out by a small group of staff (there are ~30 prescribers), hence the “density” of positive feedback was high in this area. In contrast, documentation of “decision-to-treat” time for new infections is a less common activity, carried out by a larger staff group (including bedside nurses and prescribers—approximately 300 staff); thus the “density” of positive feedback was much lower for this process. This observation suggests there may be a dose-response relationship between positive feedback and behavioral change in this setting.

Other processes improved without direct intervention (notably verbal and written review of antibiotics), which may be explained by the Hawthorne effect,^14^ or by a general increased awareness of AMS in the PICU team.

The observed reduction in antimicrobial consumption occurred despite an unexpectedly high level of the appropriate choice of initial antimicrobials, suggesting there may have been earlier termination and de-escalation of antimicrobial therapy during the project. We did not measure this activity directly, but it may have been enhanced through improved written prescriptions, in which indication and review date are documented. Visible improvements in a key process in a system may lead to increased the general awareness of the wider QI endeavor, potentially catalyzing improvements in other processes. The observed increase in the verbal and written antimicrobial review is consistent with this proposition.

The project tested an exclusively strengths-based approach to QI in a clinical environment: all interventions were designed to reinforce strengths rather than to correct weaknesses. This positive approach differs from the prevailing, deficit-based approach to improvement, and is informed by the observation that failures in healthcare are rare when compared with successes.^15^ Thus, the study of failures gives a smaller number of incidents from which to derive learning. This notion underpins the theory of Safety-II—an emerging concept in safety science.^15^ In Safety-II, safety is conceptualized as a condition in which as many things as possible go right, rather than a condition in which as few things as possible go wrong. LfE and the approach taken in this project are, in part, inspired by the Safety-II concept; and the results provide some evidence that systems can be improved through identifying and understanding strengths.

The strengths-based approach used in this project was delivered through positive feedback for HCP behaviors. Evidence from cognitive psychology and neuroscience indicates that humans can learn from both positive and negative feedback,^10^ yet the value of positive feedback is rarely recognized in healthcare. The traditional approach of learning through negative feedback following failure may reflect an innate negativity bias in which we attribute more value to lose than gain.^16^ Understanding how cognitive processes are of relevance to healthcare is the subject of a growing academic field,^17^ to which we believe this project adds meaningful data.

### Limitations

This project has several limitations. We based the methodology on improvement science, rather than a randomized controlled trial design. Some of the observed improvements may have resulted from unmeasured processes. The project was conducted in a single-center, so applicability to other settings warrants further evaluation. The post-intervention phase was limited to 3 months; a longer period of monitoring would provide greater confidence in the evaluation of longer-term effects of the intervention.

The analysis of balancing measures was limited to HCAI rates. The project team considered this measure to be the most important balancing measure for which data were readily available. Other balancing measures, such as interruptions to prescribing, medication errors, and changes in the prevalence of AMR within the organization would add more meaning to the results, but the timescale and scope of the project were inadequate to include these metrics.

We calculated antibiotic consumption from aggregate data from existing departmental databases: a recognized methodology to estimate antimicrobial usage.^18^ While this methodology minimizes the necessary data collection, it limits the results to population-level, rather than individual-level antimicrobial use. Consumption data would be enhanced through a more in-depth measurement of “days of antimicrobial therapy per patient”: a metric which was out of scope for the available resources in this project. Comparison with the matching period for the previous year was chosen in recognition of significant seasonal variation in case-mix; however, it is possible that the patient cohorts from the 2 periods differed significantly, adding an important limitation to the consumption data. Crude comparison of routinely collected demographic and patient characteristics suggests the 2 cohorts are comparable (Table 2), but this does not account for potential unmeasured differences.

## CONCLUDING SUMMARY

Positive feedback, via LfE interventions, can be used as a QI intervention to improve processes related to AMS. Not all processes were impacted equally, and there may be a dose-response effect. Future research is indicated to test this approach in other settings. This project may be replicated outside the original environment.

## ACKNOWLEDGMENTS

The institution’s statistical advisory service provided the review of antimicrobial consumption statistics.

## Supplementary Material
