# Supplementary material for: Impact of Positive Feedback on Antimicrobial Stewardship in a Pediatric Intensive Care Unit: A Quality Improvement Project
Source: Pediatr Qual Saf. 2019 Aug 30;4(5):e206. doi: 10.1097/pq9.0000000000000206 (PMC6805100; doi:10.1097/pq9.0000000000000206)
Supplement: Supplementary file 1 [file pqs-4-e206-s001.docx]

Supplementary digital content (SDC):

**Title:**

Impact of positive feedback on antimicrobial stewardship in a Paediatric Intensive Care Unit: a quality improvement project

**Authors:**

Alison S Jones MSc, Rhian E Isaac B.Pharm, Katie L Price RSCN, Adrian C Plunkett MBBS.

SDC figure 1.

Example LfE report:

| Details Of Report   \| Reported By: \| **...** \| \| --- \| --- \| \| Report Number: \| ... \| \| Date: \| **...** \| \|  \|  \| \| Details: \| **During screening for the PRAISe project you have been recognised for excellent antibiotic prescribing. You have demonstrated 3 core quality indicators in at least one antibiotic prescription in PICU:  1. Legibility / 2. clear indication / 3. clear review date or duration. These are key components of antibiotic stewardship.** \| \| Immediate Actions: \| **By continuing to highlight episodes of good practice, the rates of gold standard prescriptions are increasing. Please continue to prescribe clearly and always remember to include the review date or duration.** \| \|  \|  \| |
| --- | --- | --- | --- | --- | --- | --- | --- | --- | --- | --- | --- | --- | --- | --- |
