# Supplementary material for: Impact of Positive Feedback on Antimicrobial Stewardship in a Pediatric Intensive Care Unit: A Quality Improvement Project
Source: Pediatr Qual Saf. 2019 Aug 30;4(5):e206. doi: 10.1097/pq9.0000000000000206 (PMC6805100; doi:10.1097/pq9.0000000000000206)

Supplementary digital content (SDC):

**Title:**

Impact of positive feedback on antimicrobial stewardship in a Paediatric Intensive Care Unit: a quality improvement project

**Authors:**

Alison S Jones MSc, Rhian E Isaac B.Pharm, Katie L Price RSCN, Adrian C Plunkett MBBS.

SDC figure 2: Run chart of HCAI rates during study period, including a period of 15 months prior to study commencement:


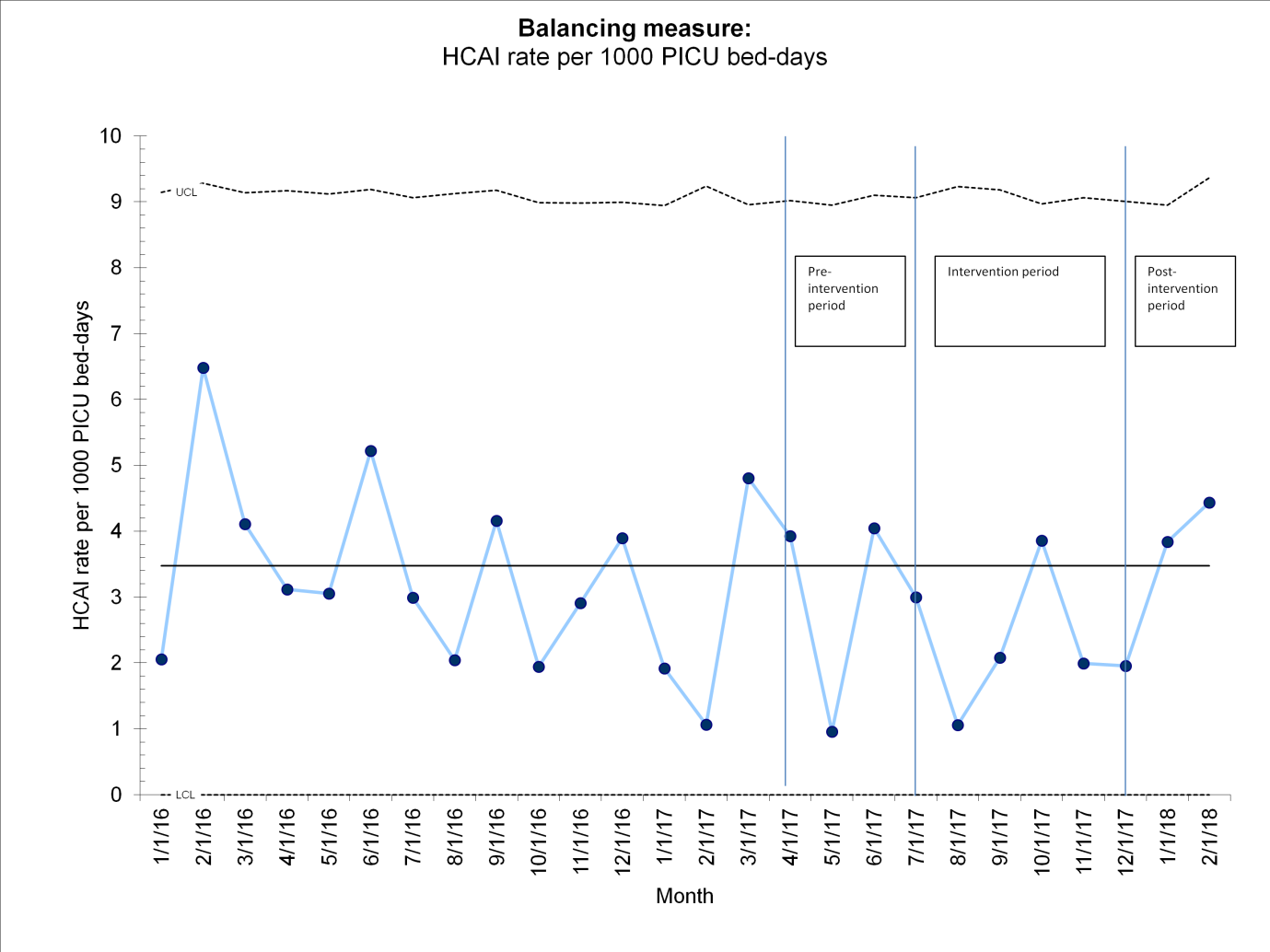

Supplement: Supplementary file 2 [file pqs-4-e206-s002.docx]
