# Supplementary material for: Impact of Positive Feedback on Antimicrobial Stewardship in a Pediatric Intensive Care Unit: A Quality Improvement Project
Source: Pediatr Qual Saf. 2019 Aug 30;4(5):e206. doi: 10.1097/pq9.0000000000000206 (PMC6805100; doi:10.1097/pq9.0000000000000206)
Supplement: Supplementary file 4 [file pqs-4-e206-s004.docx]

Supplementary digital content (SDC):

**Title:**

Impact of positive feedback on antimicrobial stewardship in a Paediatric Intensive Care Unit: a quality improvement project

**Authors:**

Alison S Jones MSc, Rhian E Isaac B.Pharm, Katie L Price RSCN, Adrian C Plunkett MBBS.

SDC Table 1: Appreciative Inquiry interview protocol:

| **Question** | **Answer / notes** |
| --- | --- |
| **Discovery:**  Please describe what happened that day?  What was it about you &/or the team that made it happen?  What were the challenges and what techniques did you use to overcome them?  How did it feel? |  |
| **Dream:**  Imagine, in 2 years’ time, that situation is routine. What has changed?  What are the smallest steps we could take to make that happen? |  |
| **Design/Destiny:**  How can we promote and share this excellent practice? |  |
| **Other comments:** |  |
