# Supplementary material for: Impact of Positive Feedback on Antimicrobial Stewardship in a Pediatric Intensive Care Unit: A Quality Improvement Project
Source: Pediatr Qual Saf. 2019 Aug 30;4(5):e206. doi: 10.1097/pq9.0000000000000206 (PMC6805100; doi:10.1097/pq9.0000000000000206)
Supplement: Supplementary file 5 [file pqs-4-e206-s005.docx]

Supplementary digital content (SDC):

**Title:**

Impact of positive feedback on antimicrobial stewardship in a Paediatric Intensive Care Unit: a quality improvement project

**Authors:**

Alison S Jones MSc, Rhian E Isaac B.Pharm, Katie L Price RSCN, Adrian C Plunkett MBBS.

SDC table 2: Improvement Ideas arising from AI Interviews with front-line Staff

| Improvement idea | Explanation | Week implemented |
| --- | --- | --- |
| A: Prescribing area improvements: | Several interviews revealed that the dedicated prescribing areas in PICU would function better if certain practical improvements were implemented. These included introduction of a working desk lamp, and improvements in functionality of the desk-top PC. | 15 |
| B: Meropenem guideline: | In response to suggestions from AI interviews, a dedicated guideline specifically for meropenem usage was developed by the project team. This was ratified by the microbiology team and disseminated by email to all prescribing staff. Hard copies were displayed in prescribing areas. | 23 |
| C: Motivational quotes from AI interviews displayed: | Several positive comments from AI interviews were identified as worthy of wider distribution. These were printed in colourful posters and displayed in high visibility staff areas. Examples include, “There is a general ethos of good prescribing in this unit”. | 26 |
| D: Project reports featured in Safety Bulletin: | Updates from the project team were circulated to all PICU staff as part of the weekly safety bulletin, in response to comments that information relating to the project is likely to inspire further improvements. | 28 |
| E: “RAG” rating antimicrobial prescriptions | One interviewee suggested color-coding the antimicrobials in the prescription chart according to a “RAG” scale – Red / Amber / Green. In conjunction with the microbiology team, all commonly used antimicrobials were classified into three bands. The red band included the broadest-spectrum antimicrobials (e.g. meropenem). Pharmacy team members annotated prescription charts accordingly. | 31 |
| F: Antimicrobial prophylaxis guide: | Interviews revealed that guidance on duration of prophylactic antimicrobials was not clear, and this prompted a redevelopment and distribution of an improved guideline, focusing specifically on the prophylactic antimicrobials. | 31 |
