# Supplementary material for: Impact of Positive Feedback on Antimicrobial Stewardship in a Pediatric Intensive Care Unit: A Quality Improvement Project
Source: Pediatr Qual Saf. 2019 Aug 30;4(5):e206. doi: 10.1097/pq9.0000000000000206 (PMC6805100; doi:10.1097/pq9.0000000000000206)
Supplement: Supplementary file 6 [file pqs-4-e206-s006.docx]

Supplementary digital content (SDC):

**Title:**

Impact of positive feedback on antimicrobial stewardship in a Paediatric Intensive Care Unit: a quality improvement project

**Authors:**

Alison S Jones MSc, Rhian E Isaac B.Pharm, Katie L Price RSCN, Adrian C Plunkett MBBS.

SDC table 3: Antimicrobials included in analysis:

| Amoxicillin |
| --- |
| Benzlypenicillin |
| Cefotaxime |
| Ceftriaxone |
| Ceftazidime |
| Cefuroxime |
| Ciprofloxacin |
| Co-amoxiclav |
| Flucloxacillin |
| Gentamicin |
| Meropenem |
| Metronidazole |
| Piperacillin-tazobactam |
| Vancomycin |
