# Supplementary material for: Impact of Positive Feedback on Antimicrobial Stewardship in a Pediatric Intensive Care Unit: A Quality Improvement Project
Source: Pediatr Qual Saf. 2019 Aug 30;4(5):e206. doi: 10.1097/pq9.0000000000000206 (PMC6805100; doi:10.1097/pq9.0000000000000206)
Supplement: Supplementary file 7 [file pqs-4-e206-s007.docx]

Supplementary data, table 4.

**Raw data for each process measure:**

**SDC, Table 4: Process measure 1a:** Appropriate selection of new antimicrobials. N=130.

Stopped after 13 weeks due to high rates.

| Week | Denominator  Number of new antimicrobials | Numerator  Number of appropriate antimicrobials | Rate (%) |
| --- | --- | --- | --- |
| 1 | 8 | 8 | 100.0 |
| 2 | 8 | 8 | 100.0 |
| 3 | 17 | 17 | 100.0 |
| 4 | 16 | 14 | 87.5 |
| 5 | 12 | 12 | 100.0 |
| 6 | 9 | 9 | 100.0 |
| 7 | 1 | 1 | 100.0 |
| 8 | 12 | 11 | 91.7 |
| 9 | 11 | 10 | 90.9 |
| 10 | 7 | 7 | 100.0 |
| 11 | 12 | 12 | 100.0 |
| 12 | 12 | 12 | 100.0 |
| 13 | 5 | 5 | 100.0 |
