# Supplementary material for: Impact of Positive Feedback on Antimicrobial Stewardship in a Pediatric Intensive Care Unit: A Quality Improvement Project
Source: Pediatr Qual Saf. 2019 Aug 30;4(5):e206. doi: 10.1097/pq9.0000000000000206 (PMC6805100; doi:10.1097/pq9.0000000000000206)
Supplement: Supplementary file 8 [file pqs-4-e206-s008.docx]

Supplementary data, table 5

**Raw data for each process measure:**

**SDC, Table 5: Process measure 1b:** appropriately selected meropenem prescriptions (for new antimicrobial episodes). N=89

| Week | Denominator  (Number of new meropenem prescriptions) | Numerator  (Number of appropriate new meropenem prescriptions) | Rate (%) |
| --- | --- | --- | --- |
| 14 | 5 | 5 | 100.0 |
| 16 | 3 | 2 | 66.7 |
| 20 | 5 | 3 | 60.0 |
| 22 | 1 | 1 | 100.0 |
| 24 | 3 | 3 | 100.0 |
| 26 | 5 | 5 | 100.0 |
| 28 | 6 | 5 | 83.3 |
| 30 | 7 | 7 | 100.0 |
| 32 | 7 | 6 | 85.7 |
| 34 | 5 | 5 | 100.0 |
| 36 | 8 | 7 | 87.5 |
| 38 | 2 | 2 | 100.0 |
| 40 | 2 | 2 | 100.0 |
| 42 | 6 | 5 | 83.3 |
| 44 | 11 | 6 | 54.5 |
| 46 | 3 | 2 | 66.7 |
| 48 | 6 | 5 | 83.3 |
| 50 | 4 | 4 | 100.0 |
