# Supplementary material for: Impact of Positive Feedback on Antimicrobial Stewardship in a Pediatric Intensive Care Unit: A Quality Improvement Project
Source: Pediatr Qual Saf. 2019 Aug 30;4(5):e206. doi: 10.1097/pq9.0000000000000206 (PMC6805100; doi:10.1097/pq9.0000000000000206)
Supplement: Supplementary file 9 [file pqs-4-e206-s009.docx]

Supplementary data, table 6

**Raw data for each process measure:**

**SDC, Table 6: Process measure 2a:** New antimicrobials administered within 1 hour of decision-to-treat. N=217.

| Week | Denominator  New antimicrobial episodes with complete documentation | Numerator  New antimicrobials administered within 1 hour of decision-to-treat | Rate (%) |
| --- | --- | --- | --- |
| 2 | 2 | 0 | 0 |
| 4 | 5 | 4 | 80 |
| 6 | 2 | 2 | 100 |
| 8 | 0 | 0 | 0 |
| 10 | 3 | 2 | 66.7 |
| 12 | 8 | 4 | 50 |
| 14 | 6 | 4 | 66.7 |
| 16 | 7 | 6 | 85.7 |
| 18 | 8 | 4 | 50 |
| 20 | 7 | 5 | 71.4 |
| 22 | 7 | 5 | 71.4 |
| 24 | 15 | 14 | 93.3 |
| 26 | 10 | 9 | 90 |
| 28 | 11 | 8 | 72.7 |
| 30 | 12 | 10 | 83.3 |
| 32 | 15 | 13 | 86.7 |
| 34 | 16 | 12 | 75 |
| 36 | 6 | 5 | 83.3 |
| 38 | 14 | 14 | 100 |
| 40 | 8 | 7 | 87.5 |
| 42 | 9 | 7 | 77.8 |
| 44 | 15 | 10 | 66.7 |
| 46 | 11 | 10 | 90.9 |
| 48 | 8 | 7 | 87.5 |
| 50 | 9 | 7 | 77.8 |
| 52 | 3 | 3 | 100 |
