# Supplementary material for: Impact of Positive Feedback on Antimicrobial Stewardship in a Pediatric Intensive Care Unit: A Quality Improvement Project
Source: Pediatr Qual Saf. 2019 Aug 30;4(5):e206. doi: 10.1097/pq9.0000000000000206 (PMC6805100; doi:10.1097/pq9.0000000000000206)
Supplement: Supplementary file 10 [file pqs-4-e206-s010.docx]

Supplementary data, table 7

**Raw data for each process measure:**

**SDC, Table 7: Process measure 2b:** documentation of decision to treat (for new antimicrobials in PICU). N=521

| Week | Denominator:  (New antimicrobial episodes) | Numerator:  (Decision-to-treat time documented) | Rate (%) |
| --- | --- | --- | --- |
| 1 | 7 | 3 | 42.9 |
| 2 | 8 | 3 | 37.5 |
| 3 | 12 | 8 | 66.7 |
| 4 | 9 | 1 | 11.1 |
| 5 | 12 | 1 | 8.3 |
| 6 | 9 | 1 | 11.1 |
| 7 | 1 | 0 | 0.0 |
| 8 | 11 | 0 | 0.0 |
| 9 | 11 | 7 | 63.6 |
| 10 | 7 | 2 | 28.6 |
| 11 | 10 | 6 | 60.0 |
| 12 | 12 | 6 | 50.0 |
| 13 | 4 | 3 | 75.0 |
| 14 | 8 | 3 | 37.5 |
| 15 | 10 | 7 | 70.0 |
| 16 | 4 | 1 | 25.0 |
| 17 | 9 | 6 | 66.7 |
| 18 | 4 | 3 | 75.0 |
| 19 |  |  |  |
| 20 | 12 | 10 | 83.3 |
| 21 | 10 | 3 | 30.0 |
| 22 | 8 | 5 | 62.5 |
| 23 | 12 | 12 | 100.0 |
| 24 | 5 | 3 | 60.0 |
| 25 | 12 | 5 | 41.7 |
| 26 | 10 | 5 | 50.0 |
| 27 | 14 | 10 | 71.4 |
| 28 | 11 | 2 | 18.2 |
| 29 | 9 | 7 | 77.8 |
| 30 | 11 | 5 | 45.5 |
| 31 | 12 | 7 | 58.3 |
| 32 | 11 | 8 | 72.7 |
| 33 | 9 | 6 | 66.7 |
| 34 | 19 | 10 | 52.6 |
| 35 | 9 | 5 | 55.6 |
| 36 | 7 | 1 | 14.3 |
| 37 | 24 | 10 | 41.7 |
| 38 | 8 | 4 | 50.0 |
| 39 | 10 | 3 | 30.0 |
| 40 | 12 | 5 | 41.7 |
| 41 | 15 | 5 | 33.3 |
| 42 | 8 | 4 | 50.0 |
| 43 | 10 | 7 | 70.0 |
| 44 | 17 | 8 | 47.1 |
| 45 | 22 | 10 | 45.5 |
| 46 | 9 | 3 | 33.3 |
| 47 | 8 | 3 | 37.5 |
| 48 | 10 | 5 | 50.0 |
| 49 | 15 | 5 | 33.3 |
| 50 | 11 | 4 | 36.4 |
| 51 | 13 | 4 | 30.8 |
