# Supplementary material for: Impact of Positive Feedback on Antimicrobial Stewardship in a Pediatric Intensive Care Unit: A Quality Improvement Project
Source: Pediatr Qual Saf. 2019 Aug 30;4(5):e206. doi: 10.1097/pq9.0000000000000206 (PMC6805100; doi:10.1097/pq9.0000000000000206)
Supplement: Supplementary file 12 [file pqs-4-e206-s012.docx]

Supplementary data, table 9

**Raw data for each process measure:**

**SDC, Table 9: Process measure 3a:** Proportion of PICU bed-days with daily written antimicrobial review. N=1539

| Week | Denominator  Number of screened patients receiving antimicrobials | Numerator  Number of screened patient records with documented antimicrobial review in previous 24 hours | Rate (%) |
| --- | --- | --- | --- |
| 1 | 32 | 10 | 31.3 |
| 2 | 43 | 10 | 23.3 |
| 3 | 39 | 13 | 33.3 |
| 4 | 30 | 20 | 66.7 |
| 5 | 46 | 11 | 23.9 |
| 6 | 35 | 10 | 28.6 |
| 7 | 26 | 10 | 38.5 |
| 8 | 30 | 9 | 30.0 |
| 9 | 33 | 4 | 12.1 |
| 10 | 38 | 17 | 44.7 |
| 11 | 26 | 7 | 26.9 |
| 12 | 29 | 13 | 44.8 |
| 13 | 25 | 6 | 24.0 |
| 14 | 36 | 11 | 30.6 |
| 15 | 34 | 16 | 47.1 |
| 16 | 24 | 7 | 29.2 |
| 17 | 34 | 14 | 41.2 |
| 18 | 25 | 8 | 32.0 |
| 19 |  | 0 |  |
| 20 | 28 | 8 | 28.6 |
| 21 | 28 | 14 | 50.0 |
| 22 | 24 | 11 | 45.8 |
| 23 | 26 | 8 | 30.8 |
| 24 | 19 | 7 | 36.8 |
| 25 | 29 | 7 | 24.1 |
| 26 | 28 | 11 | 39.3 |
| 27 | 39 | 21 | 53.8 |
| 28 | 28 | 18 | 64.3 |
| 29 | 32 | 16 | 50.0 |
| 30 | 30 | 14 | 46.7 |
| 31 | 27 | 13 | 48.1 |
| 32 | 23 | 22 | 95.7 |
| 33 | 36 | 19 | 52.8 |
| 34 | 37 | 25 | 67.6 |
| 35 | 28 | 18 | 64.3 |
| 36 | 28 | 25 | 89.3 |
| 37 | 35 | 26 | 74.3 |
| 38 | 25 | 19 | 76.0 |
| 39 | 25 | 16 | 64.0 |
| 40 | 32 | 17 | 53.1 |
| 41 | 32 | 21 | 65.6 |
| 42 | 30 | 16 | 53.3 |
| 43 | 30 | 17 | 56.7 |
| 44 | 36 | 21 | 58.3 |
| 45 | 43 | 40 | 93.0 |
| 46 | 27 | 16 | 59.3 |
| 47 | 22 | 16 | 72.7 |
| 48 | 34 | 23 | 67.6 |
| 49 | 27 | 22 | 81.5 |
| 50 | 33 | 22 | 66.7 |
| 51 | 33 | 30 | 90.9 |
