# Supplementary material for: Impact of Positive Feedback on Antimicrobial Stewardship in a Pediatric Intensive Care Unit: A Quality Improvement Project
Source: Pediatr Qual Saf. 2019 Aug 30;4(5):e206. doi: 10.1097/pq9.0000000000000206 (PMC6805100; doi:10.1097/pq9.0000000000000206)
Supplement: Supplementary file 13 [file pqs-4-e206-s013.docx]

Supplementary data, table 10

**Raw data for each process measure:**

**SDC, Table 10: Process measure 3b:** Proportion of PICU bed-day with daily verbal antimicrobial review. N=1539

| Week | Denominator  Number of screened patients receiving antimicrobials | Numerator  Number of screened patient bed spaces with verbal antimicrobial review in previous 24 hours | Rate (%) |
| --- | --- | --- | --- |
| 1 | 32 | 12 | 37.5 |
| 2 | 43 | 15 | 34.9 |
| 3 | 39 | 16 | 41.0 |
| 4 | 30 | 10 | 33.3 |
| 5 | 46 | 18 | 39.1 |
| 6 | 35 | 14 | 40.0 |
| 7 | 26 | 7 | 26.9 |
| 8 | 30 | 5 | 16.7 |
| 9 | 33 | 11 | 33.3 |
| 10 | 38 | 13 | 34.2 |
| 11 | 26 | 10 | 38.5 |
| 12 | 29 | 20 | 69.0 |
| 13 | 25 | 12 | 48.0 |
| 14 | 36 | 24 | 66.7 |
| 15 | 34 | 27 | 79.4 |
| 16 | 24 | 17 | 70.8 |
| 17 | 34 | 21 | 61.8 |
| 18 | 25 | 17 | 68.0 |
| 19 |  | 0 |  |
| 20 | 28 | 22 | 78.6 |
| 21 | 28 | 24 | 85.7 |
| 22 | 24 | 24 | 100.0 |
| 23 | 26 | 15 | 57.7 |
| 24 | 19 | 12 | 63.2 |
| 25 | 29 | 13 | 44.8 |
| 26 | 28 | 17 | 60.7 |
| 27 | 39 | 28 | 71.8 |
| 28 | 28 | 22 | 78.6 |
| 29 | 32 | 21 | 65.6 |
| 30 | 30 | 21 | 70.0 |
| 31 | 27 | 21 | 77.8 |
| 32 | 23 | 22 | 95.7 |
| 33 | 36 | 21 | 58.3 |
| 34 | 37 | 24 | 64.9 |
| 35 | 28 | 20 | 71.4 |
| 36 | 28 | 25 | 89.3 |
| 37 | 35 | 27 | 77.1 |
| 38 | 25 | 18 | 72.0 |
| 39 | 25 | 15 | 60.0 |
| 40 | 32 | 19 | 59.4 |
| 41 | 32 | 21 | 65.6 |
| 42 | 30 | 20 | 66.7 |
| 43 | 30 | 12 | 40.0 |
| 44 | 36 | 21 | 58.3 |
| 45 | 43 | 41 | 95.3 |
| 46 | 27 | 18 | 66.7 |
| 47 | 22 | 13 | 59.1 |
| 48 | 34 | 26 | 76.5 |
| 49 | 27 | 23 | 85.2 |
| 50 | 33 | 22 | 66.7 |
| 51 | 33 | 29 | 87.9 |
