# Supplementary material for: Impact of Positive Feedback on Antimicrobial Stewardship in a Pediatric Intensive Care Unit: A Quality Improvement Project
Source: Pediatr Qual Saf. 2019 Aug 30;4(5):e206. doi: 10.1097/pq9.0000000000000206 (PMC6805100; doi:10.1097/pq9.0000000000000206)
Supplement: Supplementary file 14 [file pqs-4-e206-s014.docx]

Supplementary data, table 11

**Raw data for each process measure:**

**SDC, Table 11: Process measure 4:** rate of gold standard antimicrobial prescribing. n=3731

| Week | Denominator  (number of prescriptions screened) | Numerator  (gold standard prescriptions) | % gold standard |
| --- | --- | --- | --- |
| 1 | 60 | 31 | 51.7 |
| 2 | 79 | 28 | 35.4 |
| 3 | 67 | 40 | 59.7 |
| 4 | 69 | 23 | 33.3 |
| 5 | 123 | 46 | 37.4 |
| 6 | 102 | 45 | 44.1 |
| 7 | 66 | 11 | 16.7 |
| 8 | 82 | 37 | 45.1 |
| 9 | 67 | 32 | 47.8 |
| 10 | 83 | 48 | 57.8 |
| 11 | 71 | 40 | 56.3 |
| 12 | 88 | 56 | 63.6 |
| 13 | 70 | 36 | 51.4 |
| 14 | 93 | 56 | 60.2 |
| 15 | 95 | 35 | 36.8 |
| 16 | 74 | 39 | 52.7 |
| 17 | 89 | 47 | 52.8 |
| 18 | 59 | 40 | 67.8 |
| 19 |  |  |  |
| 20 | 62 | 34 | 54.8 |
| 21 | 55 | 41 | 74.5 |
| 22 | 61 | 45 | 73.8 |
| 23 | 59 | 44 | 74.6 |
| 24 | 35 | 21 | 60.0 |
| 25 | 65 | 44 | 67.7 |
| 26 | 49 | 32 | 65.3 |
| 27 | 85 | 62 | 72.9 |
| 28 | 69 | 45 | 65.2 |
| 29 | 71 | 55 | 77.5 |
| 30 | 70 | 46 | 65.7 |
| 31 | 71 | 50 | 70.4 |
| 32 | 63 | 41 | 65.1 |
| 33 | 75 | 42 | 56.0 |
| 34 | 102 | 80 | 78.4 |
| 35 | 75 | 63 | 84.0 |
| 36 | 82 | 57 | 69.5 |
| 37 | 78 | 56 | 71.8 |
| 38 | 82 | 72 | 87.8 |
| 39 | 68 | 61 | 89.7 |
| 40 | 65 | 56 | 86.2 |
| 41 | 83 | 60 | 72.3 |
| 42 | 73 | 58 | 79.5 |
| 43 | 68 | 50 | 73.5 |
| 44 | 71 | 62 | 87.3 |
| 45 | 111 | 74 | 66.7 |
| 46 | 60 | 41 | 68.3 |
| 47 | 55 | 47 | 85.5 |
| 48 | 81 | 57 | 70.4 |
| 49 | 80 | 70 | 87.5 |
| 50 | 77 | 69 | 89.6 |
| 51 | 93 | 82 | 88.2 |
